# Determinants of high annual sickness absence in older workers: a prospective cohort study in England (Health and Employment After Fifty study)

**DOI:** 10.1136/bmjopen-2025-113474

**Published:** 2026-06-30

**Authors:** Stefania D’Angelo, Georgia Ntani, Elena Zaballa, Ira Madan, David Coggon

**Affiliations:** 1 MRC Lifecourse Epidemiology Centre, University of Southampton, Southampton, UK; 2 Occupational Health Service, Guy's and St Thomas’ NHS Foundation Trust, London, UK; 3 King’s College London School of Population Health and Environmental Sciences, London, UK

**Keywords:** Workplace, EPIDEMIOLOGY, Risk Factors

## Abstract

**Abstract:**

**Objectives:**

To quantify contributions of health- and employment-related risk factors to high annual sickness absence (HASA) among a population-based cohort of older workers in England.

**Design:**

Prospective cohort study.

**Setting:**

24 general practices geographically dispersed across England.

**Participants:**

Men and women initially aged 50–64 years recruited as part of the Health and Employment After Fifty study.

**Primary and secondary outcome measures:**

HASA, defined as a total of more than 20 days sickness absence over a 12-month interval.

**Results:**

4726 men and women were analysed, providing data on 15 333 12-month follow-up intervals. HASA was reported in 1003 (6.5%) follow-up intervals. In adjusted models, the aspects of health with the largest population-attributable fractions (PAFs) % were disabling musculoskeletal pain (25.4, 95% CI 21.2 to 29.3) and depression (11.3, 95% CI 6.1 to 16.2). Among long-term determinants of health, high body mass index had the greatest impact (PAF% 18.2, 95% CI 8.9 to 26.5). After allowance for health, high physical demands of work (PAF% 22.8) and eligibility for more generous sick pay (PAF% 25.8) made substantial contributions.

**Conclusions:**

Strategies to minimise avoidable sickness absence at older ages should prioritise: reversal of recent increases in disabling mental illness; encouragement of continued activity in workers with non-specific musculoskeletal pain, supported by modification of occupational tasks if needed; timely surgery for disabling osteoarthritis; reducing obesity; and increasing opportunities for placement in work that is less demanding physically. Further research is needed to clarify the impacts of generous sick pay on patterns of sickness absence.

**STRENGTHS AND LIMITATIONS OF THIS STUDY**

- Risk factors were determined from questionnaires completed prospectively before the high annual sickness absence (HASA) outcome, reducing potential for recall bias.
- Follow-up ceased before the onset of COVID-19, which precluded pandemic-related confounding.
- Inclusion of a wide range of potentially important determinants of high annual sickness absence among older workers.
- Data were self-reported.
- It is possible that participants were atypical of the national population in their prevalence of exposure to some risk factors and/or experience of HASA, leading to bias in estimates of population-attributable fractions.

## Introduction

 As demographic profiles evolve in consequence of reduced birth rates and increased life expectancy, the UK government is promoting work to older ages.^1^ In particular, as in many other European countries, it is raising the ages at which citizens become eligible for a state pension.^2^ However, the frequency of disabling disease rises with age, and this may impact importantly on the ability of older people to work productively, causing both sickness absence^3 4^ and early retirement.

The longitudinal Health and Employment After Fifty (HEAF) study was initiated to explore the complex inter-relation of health and work in late middle age among a sample of adults from the general population.^5^ In this paper, we use data from the HEAF study to explore the frequency and determinants of high annual sickness absence (HASA) in older workers. A companion paper examines risk factors for early long-term economic inactivity (submitted for publication).

Previous research has identified numerous factors associated with HASA in working populations, including sociodemographic characteristics and various aspects of health and employment. Mental illness, especially depression and anxiety, is a major contributor to prolonged absence from work.^6 7^ Likewise, musculoskeletal disorders have consistently been identified as leading causes of sickness absence across multiple populations.^8 9^ Long-term determinants of health such as body mass index (BMI), smoking and alcohol consumption have also been linked to sickness absence, but the extent of their contribution requires further investigation.^10 11^ Relevant workplace factors include physical demands of work, relationships with colleagues and employment conditions, all of which have been related to absence rates.^12 13^ The generosity of sick pay schemes is another possible factor, but, although some studies have found that more generous sick pay tends to be associated with higher levels of sickness absence,^14 15^ its impact on absence behaviour is complex and remains somewhat controversial.^16 17^ The relative importance of such risk factors on sickness absence specifically among older workers is less well characterised.

## Methods

### The HEAF study

The methods of the HEAF study have been reported previously.^5^ Briefly, in 2012 the Clinical Practice Research Datalink advertised the HEAF study to all practices in England contributing to its database. Practices that volunteered to assist recruitment into the HEAF study were made known to the research team and all that did so became foci of recruitment, until the required sample size was reached. In all, 24 general practices contributed to the sampling frame (from January 2013 to June 2014), offering geographical spread with practices from the South, Midlands and North of England. Practices sent an invitation to join the study with an accompanying baseline questionnaire to all men and women born between 1948 and 1964 (other than those suffering from terminal illnesses or recently bereaved). Those who agreed to take part and returned the questionnaire were subsequently asked to complete annual follow-up questionnaires over the next 6 years.

Among other things, the baseline questionnaire (online supplemental file 1) asked about sociodemographic characteristics, various aspects of health, health-related behaviours, current employment and caring commitments outside work. The first five follow-up questionnaires used identical questions to collect updated information about health, employment (including any changes of job) and caring commitments, and asked about total sickness absence in past year. The sixth annual follow-up did not collect complete information on sickness absence, and therefore was not used in the analysis for this report.

### Statistical analysis

Statistical analysis was carried out with STATA V.17.0^18^ and was based on follow-up intervals. An interval was eligible for inclusion if questionnaires had been satisfactorily completed at both its start and finish, and the participant had remained in the same job throughout its course.

### Outcome

The outcome for this report was HASA. This was ascertained from the questionnaire completed at the end of the follow-up interval and was defined as a reported total of more than 20 days sickness absence in the 12 months up to completion of that questionnaire. The reference category was no HASA over the previous 12 months.

### Risk factors

Potential risk factors were identified a priori based on existing literature on determinants of sickness absence^6^,^15^ and are listed in tables 2–4. They comprised: sex and age (both expected to influence HASA and treated as potential confounders of associations with other risk factors); sociodemographic characteristics (included mainly for descriptive purposes); aspects of health and health-related behaviours that might plausibly predispose to subsequent HASA, either directly or indirectly; aspects of work and conditions of employment that might plausibly affect the difficulty or tolerability of work in the presence of illness, thereby influencing propensity to sickness absence; and caring responsibilities outside work, which might be an encouragement to higher sickness absence. These variables were ascertained mainly from the questionnaire completed at the start of the follow-up interval under consideration, but a few, which were not expected to change greatly over the course of follow-up, and which are identified in tables 2–4, were determined from the baseline questionnaire.

Deprivation in the area of the participant’s general practice was classified using the English Index of Multiple Deprivation 2010,^19^ with scores ranging from 1 to 10. These were aggregated as ≤6 (most deprived), 7–8 and 9–10 (least deprived). Depression was defined as a score of 16 or higher on the Center for Epidemiologic Studies Depression scale.^20^ Somatising tendency was assessed through five questions from the Brief Symptoms Inventory^21^ and was defined as report that two or more of five symptoms (faintness or dizziness, pains in the heart or chest, nausea or upset stomach, trouble getting breath and hot or cold spells) had been at least moderately distressing during the past 7 days. Sleep problems were ascertained with a revised version of the Sleep Problems Scale of Jenkins *et al*.^22^ Participants reporting severe symptoms on at least one item were classified as having sleep problems. BMI was calculated from reported weight and height. Unintentional weight loss referred to loss of at least 4.5 kg in the previous 12 months, without deliberate dieting or exercise. Physical demands of work were derived from self-reported activities during a typical working day. They were classed as low where the participant reported that their job did not entail hard physical work or involve any of a specified list of activities that could be physically demanding; intermediate where they reported exposure to at least one such activity, but that their job did not involve hard physical work and heavy where they said that their job involved hard physical work. The variable ‘Difficult relationships and/or worries at work’ was derived from questions about being unfairly criticised at work, finding someone at work difficult to get along with and often worrying about work. It was classed as present if any of these applied. A job was deemed rewarding if participants experienced a general sense of achievement at work and felt appreciated in the workplace.

### Regression models

Associations of HASA with risk factors were assessed by multilevel logistic regression in which the clustering variable was the individual participant and summarised by ORs with 95% CIs. In preliminary analyses, we explored associations separately with sociodemographic characteristics, measures of current health, long-term determinants of health and work-related variables (including caring commitments). Within each category of variable, we first derived risk estimates for individual risk factors adjusted only for sex and age, followed by mutually adjusted risk estimates that accounted for all variables within the category that individually showed significant associations (lower 95% confidence limit≥1). We then constructed final multivariable models incorporating selected variables from all of the last three categories (ie, excluding the sociodemographic that had been analysed only for descriptive purposes). The rationale for the choice of variables for inclusion in the final models is set out further in the ‘Results’ section below. From the final models, we also estimated population-attributable fractions (PAFs), expressed as percentages, with associated 95% CIs.

### Patient and public involvement

Patients and/or the public were not involved in the design, or conduct, or reporting, or dissemination plans of this research.

## Results

Of the 39 359 participants invited to join the HEAF study, 8134 (20.7%) agreed and completed the baseline questionnaire, including 5728 who were in work at some point over the course of the study. However, 459 did not respond to any of the follow-up questionnaires, and a further 543 did not contribute usable information on any follow-up intervals (eg, because of changes in job). This left a total of 4726 (2289 men and 2437 women) who were included in the analysis.

Table 1 shows the distribution of participants according to the number of follow-up intervals on which they provided data, and the number of those intervals in which HASA occurred. HASA was reported in 1003 (6.5%) of the total of 15 533 follow-up intervals. Table 1 also shows the distribution that would have been expected if for each participant, the probability of HASA in any follow-up interval was equal to the overall prevalence of such absence across all follow-up intervals. Among participants who contributed data on multiple intervals, there was a tendency for HASA to recur, the frequency of HASA in two or more intervals being higher than expected.

**Table 1:** Distribution of participants according to number of follow-up intervals analysed and number of those intervals with high annual sickness absence

Number of intervals with high annual sickness absence	Number of intervals analysed				
	1	2	3	4	5
0	732 (761)	675 (688)	646 (638)	745 (710)	1149 (1017)
1	81 (52)	94 (95)	96 (132)	136 (196)	195 (351)
2		17 (3)	30 (9)	34 (20)	53 (48)
3			8 (0)	11 (1)	20 (3)
4				1 (0)	3 (0)
5					0 (0)
Total	813	786	780	927	1420

Figures in brackets are the numbers that would have been expected if for all participants, the probability of high annual sickness absence in any follow-up interval was equal to the overall prevalence of such absence across all follow-up intervals. Because of rounding, expected numbers do not always sum exactly to the column totals.

Associations of HASA with sociodemographic characteristics are summarised in table 2. After adjustment for other variables in the table, risk of HASA was significantly elevated in women (OR 1.5, 95% CI 1.2 to 1.8), at older ages (OR 1.5, 95% CI 1.1 to 2.0, for age 60+ years vs 50–54 years), among participants who were single, widowed or divorced (OR 1.3, 95% CI 1.0 to 1.6), in more deprived areas (OR 1.7, 95% CI 1.3 to 2.3, for most vs least deprived) and in people with manual and routine jobs (OR 1.5, 95% CI 1.2 to 1.9, compared with higher managerial occupations).

**Table 2:** Associations of high annual sickness absence with sociodemographic characteristics

Risk factor	Number of follow-up intervals analysed	Number (%) of follow-up intervals with HASA	Model 1* OR (95% CI)	Model 2† OR (95% CI)
Sex‡				
Male	7445	415 (5.6)	Ref	Ref
Female	8088	588 (7.3)	1.5 (1.2 to 1.8)	1.5 (1.2 to 1.8)
Age (years)				
50–54	2078	107 (5.1)	Ref	Ref
55–59	6582	423 (6.4)	1.4 (1.1 to 1.8)	1.3 (1.0 to 1.7)
60+	6873	473 (6.9)	1.6 (1.2 to 2.1)	1.5 (1.1 to 2.0)
Marital status				
Married/civil partnership/co-habiting	11 915	716 (6.0)	Ref	Ref
Single/widowed/divorced	3558	283 (8.0)	1.3 (1.1 to 1.6)	1.3 (1.0 to 1.6)
Missing	60	4 (6.7)	–	–
Level of education‡				
No qualification/school only	5072	363 (7.2)	1.4 (1.1 to 1.8)	1.1 (0.9 to 1.5)
Vocational training certificate	5057	355 (7.0)	1.5 (1.2 to 1.9)	1.2 (0.9 to 1.5)
University degree or higher	5404	285 (5.3)	Ref	Ref
General practice region‡				
London and Southeast	1094	52 (4.8)	Ref	Ref
South Central and Southwest	5141	354 (6.9)	1.7 (1.1 to 2.5)	1.3 (0.9 to 2.1)
East	3710	201 (5.4)	1.3 (0.8 to 2.0)	1.1 (0.7 to 1.7)
West Midlands	2026	149 (7.4)	1.9 (1.2 to 3.0)	1.4 (0.9 to 2.3)
Northeast and Northwest	3562	247 (6.9)	1.7 (1.1 to 2.6)	1.3 (0.8 to 2.0)
Area deprivation score‡				
≤6 (most deprived)	7325	559 (7.6)	2.0 (1.6 to 2.6)	1.7 (1.3 to 2.3)
7–8	4759	290 (6.1)	1.5 (1.1 to 1.9)	1.3 (1.0 to 1.8)
≥9	3449	154 (4.5)	Ref	Ref
Occupational social class§				
Higher managerial	6220	330 (5.3)	Ref	Ref
Intermediate	4384	295 (6.7)	1.3 (1.0 to 1.7)	1.2 (0.9 to 1.5)
Manual and routine	4692	367 (7.8)	1.8 (1.4 to 2.2)	1.5 (1.2 to 1.9)
Missing	237	11 (4.6)	–	–

* In model 1, each variable was analysed separately with adjustment only for sex and age.

† Model 2 included all variables for which results are presented with risk estimates mutually adjusted.

‡ Determined from baseline questionnaire.

§ Occupational group was determined only for intervals at the beginning of which, the participant was in work.

HASA, high annual sickness absence; Ref, reference.

Table 3 shows the relationship of HASA to various aspects of health. All of the specific health conditions that we examined were associated with higher rates of HASA, but after mutual adjustment and adjustment also for sex and age, the highest risks were for unintentional weight loss and disabling musculoskeletal pain. Risk was also elevated in those with higher BMI at baseline and in smokers, but was inversely related to reported alcohol intake at baseline.

**Table 3:** Associations of high annual sickness absence with health status and its determinants

Risk factor	Number of follow-up intervals analysed	Number (%) of follow-up intervals with HASA	Model 1* OR (95% CI)	Model 2† OR (95% CI)	Model 3† OR (95% CI)
Disabling pain in back, neck or limbs in past 12 months					
No	12 451	542 (4.4)	Ref	Ref	
Yes	3008	459 (15.3)	4.6 (3.9 to 5.4)	3.3 (2.8 to 4.0)	
Missing	74	2 (2.7)	–	–	
Depression					
No	11 510	548 (4.8)	Ref	Ref	
Yes	3940	450 (11.4)	2.8 (2.3 to 3.3)	1.6 (1.4 to 2.0)	
Missing	83	5 (6.0)	–	–	
Somatising tendency‡					
No	14 354	845 (5.9)	Ref	Ref	
Yes	964	144 (14.9)	3.5 (2.6 to 4.8)	1.7 (1.2 to 2.3)	
Missing	215	14 (6.5)	–	–	
Sleep problems					
No severe symptoms	13 176	721 (5.5)	Ref	Ref	
At least one severe symptom	2333	280 (12.0)	2.4 (2.0 to 3.0)	1.4 (1.1 to 1.7)	
Missing	24	2 (8.3)	–	–	
Unintentional weight loss					
No	14 736	853 (5.8)	Ref	Ref	
Yes	751	148 (19.7)	4.9 (3.8 to 6.4)	3.8 (2.9 to 4.9)	
Missing	46	2 (4.3)	–	–	
Walking speed					
Normal/fast	14 922	869 (5.8)	Ref	Ref	
Very slow/unable to walk	556	132 (23.7)	6.0 (4.5 to 8.2)	2.7 (2.0 to 3.7)	
Missing	55	2 (3.6)	–	–	
Any of the above health conditions					
No	8495	236 (2.8)	Ref		
Yes	6843	762 (11.1)	5.0 (4.1 to 6.0)		
Missing	195	5 (2.6)	–		
BMI (kg/m^2^)‡					
<25	5585	295 (5.3)	Ref		Ref
25–29.9	6021	362 (6.0)	1.3 (1.0 to 1.6)		1.3 (1.0 to 1.6)
30–34.9	2542	202 (7.9)	1.8 (1.4 to 2.4)		1.8 (1.4 to 2.4)
≥35	981	117 (11.9)	3.0 (2.1 to 4.3)		3.1 (2.2 to 4.4)
Missing	404	27 (6.7)	–		–
Alcohol intake per week (units per week)‡					
≤1	2864	234 (8.2)	Ref		
2–14	8427	484 (5.7)	0.7 (0.5 to 0.8)		
≥15	3009	179 (5.9)	0.7 (0.5 to 1.0)		
Missing	1233	106 (8.6)	–		
Smoking status‡					
Never smoked	8734	509 (5.8)	Ref		Ref
Ex-smoker	5141	347 (6.7)	1.2 (1.0 to 1.4)		1.1 (0.9 to 1.4)
Current smoker	1508	138 (9.2)	1.9 (1.4 to 2.6)		2.0 (1.5 to 2.7)
Missing	150	9 (6.0)	–		–

* In model 1, each variable was analysed separately with adjustment only for sex and age.

† Models 2 and 3 included sex, age and all of the variables for which results are presented (with risk estimates mutually adjusted). BMI and smoking status were not included in the same model as measures of current health as they were expected to act, at least in part, through those aspects of health.

‡ Somatising tendency, BMI, smoking and alcohol intake were determined from the baseline questionnaire.

BMI, body mass index; HASS, high annual sickness absence; Ref, reference.

Table 4 explores the association of HASA with work-related risk factors and potential personal impacts of sickness absence. Risk was elevated in participants with physically demanding, stressful and unrewarding work, those who were employed rather than self-employed, and those eligible for normal pay for a month or longer during sickness absence. However, there was no clear relationship to perceived job security in the event of prolonged illness or to caring responsibilities outside work.

**Table 4:** Associations of high annual sickness absence with job demands, stresses and rewards, and with potential personal impacts of sickness absence

Risk factor	Number of follow-up intervals analysed	Number (%) of follow-up intervals with HASA	Model 1* OR (95% CI)	Model 2† OR (95% CI)
Shift and/or night work				
Neither	12 612	768 (6.1)	Ref	Ref
Day shifts only	1653	151 (9.1)	1.6 (1.3 to 2.1)	1.3 (1.0 to 1.7)
Night work	823	68 (8.3)	1.5 (1.0 to 2.2)	1.0 (0.7 to 1.4)
Missing	445	16 (3.6)	–	–
Physical demands of work				
Low	6661	323 (4.8)	Ref	Ref
Intermediate	5263	383 (7.3)	1.6 (1.3 to 2.0)	1.7 (1.4 to 2.0)
Heavy	3554	295 (8.3)	2.3 (1.8 to 2.9)	2.3 (1.8 to 2.9)
Missing	55	2 (3.6)	–	–
Difficult relationships at work and/or frequent job worries disturbing sleep				
None	8497	437 (5.1)	Ref	Ref
Either	6780	556 (8.2)	1.8 (1.5 to 2.1)	1.4 (1.2 to 1.7)
Missing	256	10 (3.9)	–	–
Rewarding work				
Yes	13 486	792 (5.9)	Ref	Ref
No	1909	205 (10.7)	2.2 (1.7 to 2.7)	1.9 (1.5 to 2.3)
Missing	138	6 (4.3)	–	–
Type of contract				
Permanent	11 858	859 (7.2)	2.2 (1.6 to 2.9)	1.6 (1.2 to 2.2)
Temporary	792	38 (4.8)	1.2 (0.7 to 2.0)	1.4 (0.9 to 2.3)
Self-employed	2521	97 (3.8)	Ref	Ref
Missing	362	9 (2.5)	–	–
Normal pay in event of illness				
Up to 4 weeks	4293	228 (5.3)	Ref	Ref
1 month or more	7449	622 (8.4)	1.8 (1.5 to 2.2)	1.8 (1.4 to 2.2)
Not sure	2971	113 (3.8)	0.7 (0.5 to 0.9)	0.7 (0.5 to 0.9)
Missing	820	40 (4.9)	–	–
Job security in event of prolonged illness				
Secure	8820	582 (6.6)	Ref	
Insecure	6566	415 (6.3)	1.1 (0.9 to 1.3)	
Missing	147	6 (4.1)	–	
Caring responsibilities ‡				
None	12 220	765 (6.3)	Ref	
Up to 20 hours/week	2940	205 (7.0)	1.1 (0.9 to 1.4)	
20 hours/week	373	33 (8.8)	1.4 (0.8 to 2.5)	

* In model 1, each variable was analysed separately with adjustment only for sex and age.

† Model 2 included sex, age and all of the variables for which results are presented (with risk estimates mutually adjusted).

‡ Caring responsibilities were determined from the baseline questionnaire.

HASA, high annual sickness absence.

Table 5 presents results from two final regression models, giving estimates of PAFs as well as ORs. In the first model, we included sex, age, the six health conditions that were analysed in table 3 and four work-related risk factors from table 4. These variables were chosen because they had shown significant associations in the preliminary analyses and could be expected to act independently on risk of HASA. We omitted other sociodemographic variables because their impacts were likely to be mediated to a large extent through the more specific aspects of health and work that were under consideration. We excluded type of contract because its effect was expected to be principally through level of pay in the event of illness. After allowance for the other variables in the model, the measures of health that carried the highest ORs for HASA were unintentional weight loss (3.9, 95% CI 3.0 to 5.0) and disabling musculoskeletal pain (3.2, 95% CI 2.7 to 3.8), while the largest PAFs (%) were for disabling musculoskeletal pain (25.4, 95% CI 21.2 to 29.3) and depression (11.3, 95% CI 6.1 to 16.2). After allowance for health, the most important work-related risk factors were physically demanding work (PAF% 22.8, 95% CI 15.4 to 29.6) and normal pay for a month or longer during sickness absence (PAF% 25.8, 95% CI 16.6 to 34.0).

**Table 5:** Mutually adjusted associations of high annual sickness absence with selected measures of health status, determinants of health and job-related factors

Risk factor	Model 1 *		Model 2 *	
	OR (95% CI)	PAF† (95% CI)	OR (95% CI)	PAF† (95% CI)
Disabling pain in back, neck or limbs in past 12 months				
No	Ref			
Yes	3.2 (2.7 to 3.8)	25.4 (21.2 to 29.3)		
Missing	–			
Depression				
No	Ref			
Yes	1.5 (1.3 to 1.8)	11.3 (6.1 to 16.2)		
Missing	–			
Somatising tendency‡				
No	Ref			
Yes	1.6 (1.2 to 2.1)	3.7 (0.1 to 6.2)		
Missing	–			
Sleep problems				
No severe symptoms	Ref			
At least one severe symptom	1.3 (1.1 to 1.6)	4.8 (1.1 to 8.3)		
Missing	–			
Unintentional weight loss				
No	Ref			
Yes	3.9 (3.0 to 5.0)	8.4 (6.4 to 10.4)		
Missing	–			
Walking speed				
Normal/fast	Ref			
Very slow/unable to walk	2.8 (2.1 to 3.8)	6.0 (3.9 to 7.9)		
Missing	–			
BMI (kg/m^2^)‡				
<25			Ref	
25–29.9			1.2 (1.0 to 1.5)	18.2 (8.9 to 26.5)
30–34.9			1.6 (1.2 to 2.1)	
≥35			2.8 (2.0 to 3.9)	
Missing			–	
Smoking status‡				
Never smoked			Ref	
Ex-smoker			1.1 (0.9 to 1.3)	7.5 (1.0 to 13.5)
Current smoker			1.8 (1.4 to 2.4)	
Missing			–	
Physical demands of work				
Low	Ref		Ref	
Intermediate	1.6 (1.3 to 2.0)	22.8 (15.4 to 29.6)	1.7 (1.4 to 2.1)	
Heavy	1.9 (1.5 to 2.4)		2.3 (1.8 to 2.9)	
Missing	–		–	
Difficult relationships at work and/or frequent job worries disturbing sleep				
None	Ref		Ref	
Either	1.1 (0.9 to 1.3)	4.3 (– 2.6 to 10.8)	1.4 (1.2 to 1.7)	
Missing	–			
Rewarding work				
Yes	Ref		Ref	
No	1.5 (1.2 to 1.8)	4.8 (1.8 to 7.7)	1.9 (1.5 to 2.4)	
Missing	–			
Normal pay in event of illness				
Up to 4 weeks	Ref		Ref	
1 month or more	2.2 (1.8 to 2.8)	25.8 (16.6 to 34.0)	2.0 (1.6 to 2.5)	
Not sure	0.7 (0.5 to 1.0)		0.7 (0.6 to 1.0)	
Missing	–		–	

* Each model included sex, age and all of the variables for which results are presented (with risk estimates mutually adjusted).

† Summary PAF for values of the risk factor other than the reference value (see text).

‡ Somatising tendency, BMI and smoking were determined from the baseline questionnaire.

BMI, body mass index; PAF, population-attributable fraction; Ref, reference.

In the second model of table 5, we substituted BMI and smoking for the six health conditions that were in the first model. The logic for this was that effects of BMI and smoking could be mediated through several of the health conditions but were unlikely to be an important consequence of them. After allowance for sex, age and work-related variables, BMI above 25 carried a PAF% of 18.2 (95% CI 8.9 to 26.5) with an OR of 2.8 (95% CI 2.0 to 3.9) for severe obesity. The PAF% for ever having smoked was 7.5 (95% CI 1.0 to 13.5).

## Discussion

Our analysis indicates that depression and painful musculoskeletal disorders are major causes of HASA among older workers in England. Among the long-term determinants of health that we examined, high BMI had the greatest impact. When health was accounted for, workplace factors also had an important influence. In particular, high physical demands of work and eligibility for more generous sick pay made substantial contributions to the overall burden of HASA.

In interpreting these findings, it is important to consider the extent to which the study cohort was representative of the wider population, the potential for error from inaccurate ascertainment of the variables analysed, and the degree to which observed associations are likely to have been causal.

Apart from the exclusion of terminally ill individuals and those recently bereaved, the cohort was selected solely on the basis of age from the lists of multiple general practices geographically dispersed across England. Thus, those invited to take part should have been broadly representative of the national population. With a response rate of 20.7%, it is quite possible that the subset who agreed to contribute were atypical in their prevalence of exposure to risk factors and/or experience of HASA. For example, educated non-manual workers may have been over-represented in the final study sample. However, we have no reason to expect that associations between risk factors and HASA would differ systematically between responders and non-responders. Limitation of the analysis to follow-up intervals throughout which the participant remained in the same job may have led to selective exclusion of workers with long-term sickness who were in less secure employment because the absence caused them to lose their job. To the extent that this occurred, it would be expected to inflate risk estimates for eligibility to more generous sick pay, and spuriously lower those for heavy manual work. As the last of the analysed follow-up intervals ended in 2019, observations will not have been affected by COVID-19.

All of the data that we analysed were self-reported, but importantly, risk factors were determined from questionnaires completed prospectively before the HASA to which they were related. Thus, it is most likely that any errors in their ascertainment will have been non-differential with respect to the outcome, tending to bias risk estimates towards the null. Across all the follow-up intervals analysed, the distribution of reported total sickness absence by duration in days was 0: 8671 (55.8%); 1–5: 4037 (26.0%); 5–20: 1585 (10.2%); >20: 1003 (60.5%), with data missing for the remaining 237 (1.5%). Thus, the proportion of intervals in which the exact total duration was close to the cut-point of 20 days is likely to have been fairly small, making major misclassification of the outcome unlikely. More plausible is the possibility, already mentioned, that responders and non-responders differed in their prevalence of risk factors, leading to bias in estimates of PAFs. If anything, one might expect response rates to be lower among people with physically demanding work than in those with non-manual jobs, which would tend to cause underestimation of the associated PAF. The prevalence of obesity (23% of the follow-up intervals analysed) was lower than among people aged 55–64 in a recent national survey,^23^ again pointing to possible underestimation of the PAF. Conversely, if individuals eligible for more generous sick pay were over-represented in the cohort, the PAF for that variable may have been inflated.

Regarding the causality of observed associations, it is to be expected that ongoing health problems make sickness absence more likely. The measures of health that we analysed did not cover all forms of chronic illness that could predispose to sickness absence. However, we do not think that other illnesses would have spuriously inflated risk estimates for the aspects of health that we did measure. Nor is there reason to expect that they would confound associations of HASA with eligibility for sick pay. It is possible that people in physically demanding jobs came from poorer social circumstances, which increased their risk of sickness absence for unmeasured types of illness independently of the physical nature of their work. To the extent that this occurred, the observed associations of HASA with physically demanding work may not have been entirely causal.

The higher rates of HASA that we observed among women, those in manual occupations, and residents of more deprived areas are consistent with established patterns of health inequality in the UK^24 25^ and should be taken into account in strategies to reduce sickness absence. The association that we observed with older age is likely to result from the cumulative effects of ageing on health and work capacity.

The prominence of painful musculoskeletal illness as a predictor of HASA is consistent with previous research demonstrating the impact of musculoskeletal disorders on work capacity and employment outcomes.^26 27^ The PAF of 25.4% that we observed in our study is compatible with findings from other European cohorts, though comparisons are limited by differences in population characteristics and outcome definitions.^25 27^ The high OR (3.2) for musculoskeletal illness underscores its substantial impact on work attendance at an individual level among older workers.

Our finding that depression accounted for 11.3% of HASA cases aligns with growing recognition of mental health as a major driver of workplace absence. The prevalence of depression in our cohort (11.4% of follow-up intervals) is consistent with other studies of older working populations in the UK.^25 28^ The relationship between depression and work is probably bidirectional, with workplace pressures and job demands contributing to depression, while depression itself impairs work capacity and attendance.^29 30^

The strong association between unintentional weight loss and HASA (OR 3.9) is likely to reflect the presence of serious underlying medical conditions such as cancer and cardiorespiratory disease that we did not ascertain specifically. While unintentional weight loss had a relatively modest PAF (8.4%), the high OR suggests that it serves as an important marker of severe illness requiring medical attention and potentially prolonged absence from work.

The substantial contribution of physical work demands to HASA (PAF 22.8%) highlights the particular vulnerability of older workers in physically demanding occupations. Age-related declines in physical capacity, combined with the cumulative effects of occupational exposures, may render older workers increasingly susceptible to injury and illness in physically demanding jobs.^31 32^ Data from the Labour Force Survey for Great Britain suggest that during 2022–2025, an estimated 1.81 days per full-time equivalent worker were lost from work among people aged 55 years and older because of illness or injury which participants believed had been caused or made worse by work compared with 1.2 days per person at all ages.^33^ And even where work does not cause an illness, the associated disability will often be more of a handicap in physically demanding jobs. For example, sedentary office work may be possible with impaired mobility that would preclude jobs which entail a lot of walking. Our finding has implications for occupational health strategies, suggesting a more frequent need for job modification in older manual workers when health problems occur.

The association of more generous sick pay with higher rates of HASA (PAF 25.8%) raises complex questions about the relationship between social protection and absence behaviour. While generous sick pay is desirable to protect workers’ financial security during illness, our findings suggest that it may also encourage longer absence. It is unclear to what extent this longer absence is beneficial for workers’ health, and whether such benefits, together with the security that is engendered by generous sick pay, are sufficient to justify its costs in lost productivity.

Our findings suggest that in older workers, job stress, as evidenced by report of difficult personal relationships with colleagues or frequently worrying about work, is less important as a driver of HASA than the physical demands of work and financial aspects of employment.

High BMI appeared to contribute importantly to HASA (PAF 18.2%), risk being increased almost threefold in individuals with severe obesity (BMI≥35). This is consistent with the well-established associations of obesity with various health conditions that affect work capacity, including musculoskeletal disorders, cardiovascular disease and diabetes.^34 35^ It highlights the potential value of interventions to reduce obesity as a way of increasing productivity.

The more modest contribution of smoking to the overall burden of HASA (PAF 7.5%) reflects the relatively low prevalence of smoking in our cohort (9.7%). The adjusted OR for current smokers (1.8) was similar to that for obesity and could be expected, given the well-documented health impacts of tobacco use. The inverse relationship between alcohol consumption and HASA, with highest risk among those consuming≤1 unit per week, may be explained by reverse causation, whereby individuals with chronic health conditions reduce their alcohol intake.

### Implications

Some sickness absence is both inevitable and appropriate. The health problems that cause it cannot be totally eliminated, and the resultant disability and requirements for treatment make performance of a person’s job unreasonable or totally impossible. The challenge is to minimise avoidable and unnecessary sickness absence. Our findings point to several priorities in pursuit of this goal for older workers in the UK.

First is to reduce the prevalence of disabling depression and musculoskeletal pain. The approach to depression should start with attempts to identify and reverse causes of the marked rise in disabling mental illness which has occurred among people of working age over recent decades.^25^ It is possible that these lie more in culturally-driven changes in perceptions of mental illness than in a more stressful environment. Musculoskeletal diseases such as hip and knee osteoarthritis occur quite frequently in older workers. Although surgical treatment can reduce associated disability, research is needed to clarify the impacts of timely surgery on subsequent work participation. Studies have shown that occupational advice to patients regarding timing to return to work after surgery is limited.^34 36^ An important ongoing initiative in this area is the OPAL trial, which is testing whether structured advice delivered in hospital before and after hip or knee replacement promotes earlier and more successful return to work.^35^ As at younger ages, strategies to reduce incapacity from non-specific musculoskeletal pain in the back and arm should focus on modifying occupational tasks to make them easier for those with symptoms, while encouraging continued physical activity.^37^,^39^ Trials of ergonomic interventions suggest that any benefits in primary prevention are modest.^40^

A second opportunity may lie in reducing obesity. This is already a priority for public health, and the recent emergence of effective pharmacological treatments offers the prospect of more successful interventions.^41^ Our findings support the case for including sickness absence as an outcome in trials that are carried out among people of working age. In addition, there may be benefits from programmes in the workplace to encourage weight management by physical activity and healthier eating,^23^ but their cost-effectiveness needs to be established.

Also in the workplace, consideration should be given to the placement of older workers. Where there is flexibility, it will often be better if older workers are assigned to jobs that are less physically demanding, especially if they have objectively diagnosable health problems such as cardiorespiratory disease or large joint osteoarthritis which can compromise ability to do heavier work.

Further research is needed to assess patterns of sickness absence in workers eligible for generous sick pay as compared with those in similar jobs who are not. Can higher rates of sickness absence in the former be confirmed, and if so, what types of illness account for the excess, and does the difference lie in the number or absence episodes, their duration or both?

Strategies such as these should help in efforts to optimise work at older ages, for the benefit both of individual workers and society more widely.

## Supplementary material

10.1136/bmjopen-2025-113474 online supplemental file 1

## Data Availability

Data are available upon reasonable request.
