# Supplementary material for: Determinants of high annual sickness absence in older workers: a prospective cohort study in England (Health and Employment After Fifty study)
Source: BMJ Open. 2026 Jun 30;16(6):e113474. doi: 10.1136/bmjopen-2025-113474 (PMC13331197; doi:10.1136/bmjopen-2025-113474)
Supplement: Supplementary file 2 [file bmjopen-2025-113474.reviewer_comments.pdf]

PEER REVIEW HISTORY

BMJ Open publishes all reviews undertaken for accepted manuscripts. Reviewers are asked to complete a checklist review form and are provided with free text boxes to elaborate on their assessment. These free text comments are reproduced below.

ARTICLE DETAILS

Title (Provisional)

Determinants of high annual sickness absence in older workers: a prospective cohort study in England (Health and Employment After Fifty study)

Authors

D'Angelo, Stefania; Zaballa, Elena; Ntani, G; MADAN, IRA; Coggon, David

---

VERSION 1 - REVIEW

**Reviewer**	**1**
**Name**	**Pickett, William**
**Affiliation**	**Queen's University, Public Health Sciences**
**Date**	**25-Jan-2026**
**COI**	**None**

Thank you for the opportunity to review this original cohort study, focused upon determinants of long-term absences from work in a British cohort of workers. There is great value in the study question and this research opportunity, and the article clearly has a number of important strengths and potentially represents a valuable contribution to the associated literature. Here are some critical comments for your consideration.

Representativeness. On several occasions throughout the article, the authors addressed the representativeness of the cohort data, both in terms of how representative that prevalence estimates would be with such a low participation rate, as well as the possibility that effect estimates would be biased (which is less likely). The study does suffer to a degree from a very low baseline response rate (20.7%), and is encumbered by further exclusions (non-responders, job changers, those leaving work). Because the major study findings focused on attributable fractions (which in part are based on prevalence levels), it is likely that the main measures of impact are subject to selection bias. Additional discussion of representativeness and the potential biases introduced due to lack of participation would be helpful. In addition, if it is possible to compare respondents and non-respondents in any quantitative analysis, that would add to the robustness of your findings and their interpretation.

Additional detail on the practices involved in this study. While some descriptive data are available in Table 2 about the distribution of participants by geographic region, it would be

helpful to an outside reader to know about the sample and its context. In addition, in the modelling that was produced, with participants nested in practices, it would be important to know if the effect of clustering was accounted for during its conduct.

Causal inference. Most of the interpretation of the study findings with respect to causation focuses in on the longitudinal nature of the study, and temporal aspects of causation. It would be helpful to the article and its interpretation if there was a broader discussion of the evidence that this study brings to the field and study question(s) in terms of causal relationships, that considers other criteria for causation.

Temporality. Given that this was a longitudinal analysis and study where key exposures were collected prior to the study outcomes, it does make sense that the authors have some confidence in the temporality of the relationships, with reverse causation unlikely. However, if a worker had an underlying illness that was undetected clinically in the early part of their involvement, it is possible that a sickness absence led to the detection of that illness – a subtle form or reverse causation. This could perhaps be recognized in the discussion, with more nuanced discussion of these temporal effects.

Need for a conceptual model. While the basic premise of the analysis is excellent, and the exploration of a number of potential factors that could lead to long-term sickness absence is sensible, the analysis would benefit from a conceptual model (in prose or a figure) that highlights the main etiological relationships under study, potential confounders, and the pathways that are implied through the analysis. As it stands, most of the relationships being examined were considered in light of a small, standard set of confounders that might vary by exposure type, effect modification was not considered at all, and the analysis lacks an in-depth description of its theoretical basis. The article would be strengthened by having one or more conceptual models, to strengthen its theoretical underpinnings.

Model building strategies. Little information is provided in the methods section to allow the reader to fully assess how models were built, and the methodological steps taken to come up with precise estimates of effect. Additional detail would be helpful.

Presentation format (methods and results). The presentation of the analysis is unusual in that the Results section contains necessary methods that perhaps are best presented in the Methods section, making this a more confusing read than usual. Perhaps the writing and order of presentation of these details could be revisited.

Implications. The study findings clearly have a number of implications, both occupationally and clinically. However, the suggestions that were provided seemed to be driven entirely by the data and were quite anecdotal, and cited in the absence of high level evidence from evaluative studies. More depth of evidence is needed to support many of the implications that were provided for the reader. Nothing that was written is necessarily “wrong”; however, the recommendations provided lacked empirical or other evidence that came from the extant literature.

**Reviewer**	**2**
**Name**	**Mustard, Cameron**
**Affiliation**	**Institute for Work and Health**
**Date**	**06-Feb-2026**
**COI**	**None**

BMJ Open 2025-113474

Determinants of high annual sickness absence in older workers: findings from the Health and Employment After Fifty (HEAF) study

This manuscript reports analysis of the longitudinal HEAF cohort to identify lifestyle, health-related and employment-related factors associated with high annual sickness absence (a total of more than 20 days of sickness absence in the previous 12 months, termed HASA). The analytic sample comprised 4,726 participants aged 50-64 at cohort inception who were in work at some point over the four year follow-up period and who contributed 15,500 12 month followup intervals. The incidence of HASA was approximately 6.5%.

Overall, the manuscript is appropriately organized, parsimonious and well-written. The analytic sample is adequately powered and the anaytic methods appear appropriate.

Primary findings reported in the manuscript include an elevated risk of HASA among women, participants at older ages, residents of more deprived areas, and workers in manual occupations. Health-related risk factors for HASA were dominated by disabling musculoskeletal pain, depression, higher BMI, and unintentional weight loss. Work-related factors associated with HASA were physically demanding work and generosity of entitlement for sick pay benefits.

As the introduction of the manuscript notes, sociodemographic, health and employment-related risk factors for sickness absence are generally understood to be well established. The rationale for conducting this analysis was to provide a specific focus on older adults with active labour force participation. Additionally, the manuscript makes a useful contribution by estimating population attributable fractions for the most substantial risk factors.

I have no substantial concerns with the rationale, methods and interpretation presented in this manuscript. I would invite the authors to consider the following suggestions.

I found the manuscript provided limited information to assess the reliability and validity of the self-reported measure of sickness absence. To aid the reader in assessing the representativeness of the sample, I'd invite the authors to consider including a supplemental table that reports the frequency distribution of sickness absence across the four data

collection categories (no absence, any absence, and stratified by < five days absence, 5-20 days absence and >20 days absence). Inclusion of denominator counts would aid the reader in estimating incidence and average days lost per worker. These values can easily be compared to routine statistics provided by the UK labour force survey.

I found myself disagreeing with the authors' perspective in the introduction that 'the generosity of sick pay schemes is another possible risk factor, but its impact on absence behavior is complex and somewhat controversial'. In my view, sick pay scheme entitlements have been well-established as associated with more sickness absence days (see the following: "Groups with the highest rates of sickness absence in 2024 included women, older workers, those with longterm health conditions, people working part-time, people working in the public sector and people working in elementary occupations. Office for National Statistics (ONS), released 4 June 2025, ONS website, article, Sickness absence in the UK labour market: 2023 and 2024). I'd encourage the authors to consider describing this finding as confirmatory, rather than novel.

As a final observation, I'd encourage the authors to revisit the discussion of sickness absence attributed to work-related health conditions (p10/line 29). The UK Health & Safety Executive estimates approximately 40 million sickness absence days annually among UK workers, which represents approximately 25% of the total sickness absence days recorded by the UK LFS in 2024 (148 million, all ages). Specifically noting the burden of work-related ill health as a component of population sickness absence would strengthen the manuscript.

VERSION 1 - AUTHOR RESPONSE

Reviewer: 1
Prof. William Pickett, Queen's University
Comments to the Author:
Thank you for the opportunity to review this original cohort study, focused upon determinants of long-term absences from work in a British cohort of workers. There is great value in the study question and this research opportunity, and the article clearly has a number of important strengths and potentially represents a valuable contribution to the associated literature. Here are some critical comments for your consideration.

Thank you for these positive comments. We should point out a minor misunderstanding. As set out in paragraph 2 of the Methods section, our questionnaires asked about "total sickness absence in the past year", and not "long-term absences". In theory, high annual sickness absence could in some cases have resulted from multiple absences of only one or two days, although we would expect it mainly to reflect longer term spells of absence. As indicated in the opening paragraph of the Introduction, our interest was in absence that impacts importantly on productivity, for which high total absence was the most relevant available measure.

Representativeness. On several occasions throughout the article, the authors addressed the representativeness of the cohort data, both in terms of how representative that prevalence estimates would be with such a low participation rate, as well as the possibility that effect estimates would be biased (which is less likely). The study does suffer to a degree from a very low baseline response rate (20.7%), and is encumbered by further exclusions (non-responders, job changers, those leaving work). Because the major study findings focused on attributable fractions (which in part are based on prevalence levels), it is likely that the main measures of impact are subject to selection

bias. Additional discussion of representativeness and the potential biases introduced due to lack of participation would be helpful. In addition, if it is possible to compare respondents and non-respondents in any quantitative analysis, that would add to the robustness of your findings and their interpretation.

As the reviewer notes, we acknowledge the low baseline response rate and already discuss at some length its potential implications for interpretation (paragraphs 2-5 of Discussion). In paragraph 3 of the Discussion, we say "With a response rate of 20.7%, it is quite possible that the subset who agreed to contribute were atypical in their prevalence of exposure to risk factors and/or experience of HASA. For example, educated non-manual workers may have been over-represented in the final study sample", and "Limitation of the analysis to follow-up intervals throughout which the participant remained in the same job may have led to selective exclusion of workers with long-term sickness who were in less secure employment because the absence caused them to lose their job. To the extent that this occurred, it would be expected to inflate risk estimates for eligibility to more generous sick pay, and spuriously lower those for heavy manual work." Then, in the next paragraph we say "More plausible is the possibility, already mentioned, that responders and non-responders differed in their prevalence of risk factors, leading to bias in estimates of population attributable fractions (PAFs). If anything, one might expect response rates to be lower among people with physically demanding work than in those with non-manual jobs, which would tend to cause underestimation of the associated PAF." We have now added "The prevalence of obesity (23% of the follow-up intervals analysed) was lower than among people aged 55-64 in a recent national survey (23), again pointing to possible underestimation of the PAF. Conversely, if individuals eligible for more generous sick pay were over-represented in the cohort, the PAF for that variable may have been inflated."

Unfortunately, we are unable to compare characteristics of responders and non-responders at baseline, as the initial mailing was carried out by the practices and the research team received information only about those individuals who consented to take part and returned a baseline questionnaire.

Additional detail on the practices involved in this study. While some descriptive data are available in Table 2 about the distribution of participants by geographic region, it would be helpful to an outside reader to know about the sample and its context. In addition, in the modelling that was produced, with participants nested in practices, it would be important to know if the effect of clustering was accounted for during its conduct.

Further information about the study sample and its context can be found in reference 5, which we cite in the first sentence of the Methods section. That paper explains that:

"In 2012, the CPRD advertised the HEAF study to all practices in England contributing data to its database. (The CPRD collects data also from Scotland, Wales and Northern Ireland, but geographical restriction was employed to allow later linkage with English databases that record hospital inpatient and outpatient care (Hospital Episode Statistics), as well as mortality and cancer incidence (Health and Social Care Information Centre)). Practices that volunteered to assist recruitment into the HEAF study were made known to the research team and all that did so

become foci of recruitment, until the target sample size was met. In all, 24 general practices finally contributed to the sampling frame (during Jan 2013 to June 2014). These offered a good geographical spread, with recruitment from the South, Midlands and North of England (Fig. 1). (There was no requirement that the distribution of respondents' occupations should be nationally representative, but geographical dispersion was deemed desirable as unemployment rates and patterns of illness behaviour and consulting are liable to vary between regions.)"

We have now added to the first paragraph of the Methods section that the 24 practices were "contributing to the Clinical Practice Research Database", but we feel that further detail is best left for readers to access through the reference.

In our analysis, we used multi-level modelling to allow for possible clustering within individuals but had no reason to expect important clustering by general practice.

Causal inference. Most of the interpretation of the study findings with respect to causation focuses in on the longitudinal nature of the study, and temporal aspects of causation. It would be helpful to the article and its interpretation if there was a broader discussion of the evidence that this study brings to the field and study question(s) in terms of causal relationships, that considers other criteria for causation.

The importance of temporality in interpretation is not so much the plausibility that it gives to the causality of observed associations, but rather its implications regarding possible recall bias. Thus, in paragraph 4 of the Discussion, we say "importantly, risk factors were determined from questionnaires completed prospectively before the HASA to which they were related. Thus, it is most likely that any errors in their ascertainment will have been non-differential with respect to the outcome, tending to bias risk estimates towards the null."

Conclusions about causation depend more upon the consistency of observed associations with other relevant research (making chance less likely as an explanation), the plausibility of potential underlying causal mechanisms and the confidence with which one can exclude confounding by other known or suspected causes of the outcomes under study. These are considered at some length in paragraphs 5 to 14 of the Discussion.

We prefer not to use formulaic approaches to causal inference based on the so-called Bradford-Hill criteria, which were not proposed for that purpose.

Temporality. Given that this was a longitudinal analysis and study where key exposures were collected prior to the study outcomes, it does make sense that the authors have some confidence in the temporality of the relationships, with reverse causation unlikely. However, if a worker had an underlying illness that was undetected clinically in the early part of their involvement, it is possible that a sickness absence led to the detection of that illness – a subtle form or reverse causation. This could perhaps be recognized in the discussion, with more nuanced discussion of these temporal effects.

As explained above, we do not invoke temporality as a strong argument for observed associations being causal (although we recognise that a cause must precede its effect). Moreover, we are unsure about the reviewer's argument about possible reverse causation. If sickness absence during a given follow-up period provided the first indication of an illness, then that illness could not have been reported in the questionnaire at the start of the relevant follow-up period.

Need for a conceptual model. While the basic premise of the analysis is excellent, and the exploration of a number of potential factors that could lead to long-term sickness absence is sensible, the analysis would benefit from a conceptual model (in prose or a figure) that highlights the main etiological relationships under study, potential confounders, and the pathways that are implied through the analysis. As it stands, most of the relationships being examined were considered in light of a small, standard set of confounders that might vary by exposure type, effect modification was not considered at all, and the analysis lacks an in-depth description of its theoretical basis. The article would be strengthened by having one or more conceptual models, to strengthen its theoretical underpinnings.

A full conceptual model would be large and complex, and we do not think it would assist many readers when assessing the validity and implications of our findings. However, we have expanded the text in the section of the Methods on risk factors to say that they "comprised: sex and age (both expected to influence HASA and treated as potential confounders of associations with other risk factors); sociodemographic characteristics (included mainly for descriptive purposes); aspects of health and health-related behaviours that might plausibly predispose to subsequent HASA, either directly or indirectly; aspects of work and conditions of employment that might plausibly affect the difficulty or tolerability of work in the presence of illness, thereby influencing propensity to sickness absence; and caring responsibilities outside work, which might be an encouragement to higher sickness absence."

We did not explore potential effect modifiers because we had no a priori reason to expect that any measured variable would importantly modify associations with those in the final model presented in Table 5.

Model building strategies. Little information is provided in the methods section to allow the reader to fully assess how models were built, and the methodological steps taken to come up with precise estimates of effect. Additional detail would be helpful.

As should be clear from their titles and content, in Tables 2 to 4 respectively we looked at associations with sociodemographic characteristics, health-related variables, and aspects of work and caring responsibilities. In Tables 2 and 4, we looked at associations when each variable was analysed separately with adjustment only for sex and age, and then from a model which included all of the risk factors in the table. In Table 3, we followed a similar approach except that we used two models with mutual adjustment. One (Model 2) included five potentially independent measures of health. The second included BMI and smoking status. We did things in this way because BMI and smoking might be expected to act, at least in part, through one or more of the measures of current health. Alcohol intake was omitted because it did not show associations in the analysis adjusted only for sex and age. We have now added a sentence in the paragraph of the Methods section on regression models, saying "Within each category of variable, we derived risk estimates for individual risk factors adjusted only for sex and age, and also mutually adjusted risk estimates". We have also expanded the footnotes to Table 3 to explain that "BMI and smoking status were not included in the same model as measures of current health as they were expected to act, at least in part, through those aspects of health."

The rationale for the final models in Table 5 is set out in some detail in the last two paragraphs of the Results section.

The statistical precision of effect estimates is reflected in associated confidence intervals, which were derived by the statistical methods that we describe in the Methods section.

Presentation format (methods and results). The presentation of the analysis is unusual in that the Results section contains necessary methods that perhaps are best presented in the Methods section, making this a more confusing read than usual. Perhaps the writing and order of presentation of these details could be revisited.

We agree that the order of presentation is unusual, and thought hard before fixing on the approach which we adopted. Our reasons were that presenting a long list or table of all the risk factors analysed as part of the Methods section would have led to substantial duplication of information which needed to be included in the Results section, making the paper more unwieldy. Moreover, the rationale for the inclusion of variables in the final Table could only be fully explained once earlier results had been presented. We still feel that this is justified. However, to make the paper easier to follow, we have added a sentence in the paragraph of the Methods section describing regression models, saying "Within each category of variable, we derived risk estimates for individual risk factors adjusted only for sex and age, and also mutually adjusted risk estimates".
We would be happy to change the order of presentation if the editors are not satisfied with the current format.

Implications. The study findings clearly have a number of implications, both occupationally and clinically. However, the suggestions that were provided seemed to be driven entirely by the data and were quite anecdotal, and cited in the absence of high level evidence from evaluative studies. More depth of evidence is needed to support many of the implications that were provided for the reader. Nothing that was written is necessarily "wrong"; however, the recommendations provided lacked empirical or other evidence that came from the extant literature.

In reporting new primary research, it is important to consider the implication of its findings for decisions in policy, practice or prioritising further research. If a study did not influence decision-making in any way, then it would not have been worth conducting. By definition, implications will be driven by the study findings but should also take into account relevant evidence from other sources. The depth in which evidence from other sources is cited when drawing conclusions and making recommendations will depend on the nature of those conclusions/recommendations. A proposal that a possibility should be considered, or that a specific line of research might be worth pursuing, does not require such full elaboration of relevant evidence as an unequivocal recommendation for a change in policy or practice (which would normally require separate systematic review).

In our Discussion, we highlight pointers which we believe our research provides to future priorities in trying to minimise avoidable and unnecessary sickness absence in older workers.

Our recommendation to try to reduce the prevalence of depression hardly seems controversial. We have cited evidence that the important contribution of depression to HASA in our study sample is consistent with other studies of older working populations in the UK, and our recommendation to focus initially on trying to "identify and reverse causes of the marked rise in disabling mental illness which has occurred among people of working age over recent decades" is a suggestion, which we feel

entitled to make (with evidence for the rise cited). The suggestion that the causes may "lie more in culturally-driven changes in perceptions of mental illness than in a more stressful environment" is clearly speculative, but we think an important possibility to consider, and again a suggestion that we are entitled to put forward.

The importance of musculoskeletal illness as a driver of HASA, seems uncontroversial, and in discussing possible approaches to its management, we cite evidence mainly from major trials and systematic reviews.

In regard to obesity, we say only that "a second opportunity <u>may</u> lie in reducing obesity", pointing out that is already a priority for public health. We do not think anyone could reasonably claim that this position runs contrary to the current balance of evidence, and our claim that "our findings support the case for including sickness absence as an outcome in trials that are carried out among people of working age" seems fair comment. We are not saying that it should be included as an outcome – only that our findings support the case for that. We do not claim that there will be benefits from programmes in the workplace to encourage weight management by physical activity and healthier eating but suggest that their cost-effectiveness needs to be established.

Our suggestion that "consideration should be given to the placement of older workers" is intended as a broad pointer to future thinking. Our statement that "Where there is flexibility, it will often be better if older workers are assigned to jobs that are less physically demanding, especially if they have objectively diagnosable health problems such as cardiorespiratory disease or large joint osteoarthritis which can compromise ability to do heavier work" accords with current occupational health thinking.

Our recommendation for further research that might help to clarify the contribution of generous sick pay to sickness absence seems reasonable given the controversy in the literature that we reference in our Introduction and the limitations of what can be gleaned from our own study.

Reviewer: 2
Prof. Cameron Mustard, Institute for Work and Health
Comments to the Author:
BMJ Open 2025-113474
Determinants of high annual sickness absence in older workers: findings from the Health and Employment After Fifty (HEAF) study

This manuscript reports analysis of the longitudinal HEAF cohort to identify lifestyle, health-related and employment-related factors associated with high annual sickness absence (a total of more than 20 days of sickness absence in the previous 12 months, termed HASA). The analytic sample comprised 4,726 participants aged 50-64 at cohort inception who were in work at some point over the four year follow-up period and who contributed 15,500 12 month followup intervals. The incidence of HASA was approximately 6.5%.

Overall, the manuscript is appropriately organized, parsimonious and well-written. The analytic sample is adequately powered and the anaytic methods appear appropriate.

Thank you for your positive comments about our paper. We note that this reviewer did not consider our choice to place some of the description of statistical model-building in the Results section to be inappropriate.

Primary findings reported in the manuscript include an elevated risk of HASA among women, participants at older ages, residents of more deprived areas, and workers in manual occupations. Health-related risk factors for HASA were dominated by disabling musculoskeletal pain, depression, higher BMI, and unintentional weight loss. Work-related factors associated with HASA were physically demanding work and generosity of entitlement for sick pay benefits.

As the introduction of the manuscript notes, sociodemographic, health and employment-related risk factors for sickness absence are generally understood to be well established. The rationale for conducting this analysis was to provide a specific focus on older adults with active labour force participation. Additionally, the manuscript makes a useful contribution by estimating population attributable fractions for the most substantial risk factors.

I have no substantial concerns with the rationale, methods and interpretation presented in this manuscript. I would invite the authors to consider the following suggestions.

I found the manuscript provided limited information to assess the reliability and validity of the self-reported measure of sickness absence. To aid the reader in assessing the representativeness of the sample, I'd invite the authors to consider including a supplemental table that reports the frequency distribution of sickness absence across the four data collection categories (no absence, any absence, and stratified by < five days absence, 5-20 days absence and >20 days absence). Inclusion of denominator counts would aid the reader in estimating incidence and average days lost per worker. These values can easily be compared to routine statistics provided by the UK labour force survey.

Thank you for your comment. Across all the follow-up intervals analysed, the distribution of reported total sickness absence by duration in days was 0: 8,671 (55.8%), 1-5: 4,037 (26.0%), 5-20: 1,585 (10.2%), >20 1,003 (6.5%) with data missing for the remaining 237 (1.5%) Thus, the proportion of intervals in which the exact total duration was close to the cut-point of 20 days is likely to have been fairly small, making major misclassification unlikely. We have expanded paragraph 4 of the Discussion to include this information.

I found myself disagreeing with the authors' perspective in the introduction that 'the generosity of sick pay schemes is another possible risk factor, but its impact on absence behavior is complex and somewhat controversial'. In my view, sick pay scheme entitlements have been well-established as associated with more sickness absence days (see the following: "Groups with the highest rates of sickness absence in 2024 included women, older workers, those with longterm health conditions, people working part-time, people working in the public sector and people working in elementary occupations. Office for National Statistics (ONS), released 4 June 2025, ONS website, article, Sickness absence in the UK labour market: 2023 and 2024). I'd encourage the authors to consider describing this finding as confirmatory, rather than novel.

Thank you for your thoughtful comment. The ONS report on sickness absence cited by the reviewer describes differences in sickness absence rates between private and public sector employees, the latter of whom typically have more generous sick pay arrangements. However, these data are descriptive and do not directly examine sick pay generosity as an independent risk factor for sickness absence. There is good evidence that sickness absence is subject to cultural influences, and it may be that higher rates in the public sector are driven more by culture than generosity of sick pay. To clinch the case, one would like to see consistent associations across a range of organisations in the private sector.

In light of this comment, we have revised the relevant sentence in the Introduction, to acknowledge that there is evidence suggesting an association between sick pay generosity and higher sickness absence rate. At the same time, we note that the strength and interpretation of the association remain debated.

As a final observation, I'd encourage the authors to revisit the discussion of sickness absence attributed to work-related health conditions (p10/line 29). The UK Health & Safety Executive estimates approximately 40 million sickness absence days annually among UK workers, which represents approximately 25% of the total sickness absence days recorded by the UK LFS in 2024 (148 million, all ages). Specifically noting the burden of work-related ill health as a component of population sickness absence would strengthen the manuscript.

Thank you for your comment. We do not anywhere refer to "work-related health conditions" in our report (although we do mention work-related risk factors), and we find the concept of "work-related illness" unhelpful. The HSE estimate of 40 million sickness absence days annually in UK workers from such illness presumably is based on reports of absence for illness which the individual considers to be caused or exacerbated by work. However, for most disorders caused by work, the link to occupation cannot be established with confidence in the individual case, even by an experienced clinician with access to good diagnostic facilities. For example, suppose a given exposure to occupational kneeling has been shown epidemiologically to increase the risk of knee osteoarthritis leading to joint surgery with a relative risk of 1.5. It follows that on average, for every three cases with the occupational exposure, one will have developed the disease because of the exposure, and two would have developed it even in the absence of the exposure. However, no-one can say which of the three cases was caused by work.

Our concern in this report is not whether health problems are caused by work, but whether they impinge on capacity to work, leading to high annual sickness absence.

VERSION 2 - REVIEW

**Reviewer**	**1**
**Name**	**Pickett, William**
**Affiliation**	**Queen's University, Public Health Sciences**
**Date**	**06-Mar-2026**
**COI**	

Thank you for your detailed and thoughtful response to the issues that I raised upon initial review. I know that it is challenging at times to respond to every nuanced observation made about your work, especially when you are close to it, and I was just trying to provide an honest assessment of its potential strengths and flaws from my viewpoint.

Most of your responses are detailed and thoughtful, and I have respect for your counter-arguments. Here are the remaining ones that didn't sit well with me.

It is not an unreasonable thing to ask for more detail on your study base, and reference to another article for that information ... while technically okay .. puts the onus on the reader to find that information and then also assumes that they have access to that article. I did not think it was an unreasonable thing to ask for, and disagree with your response that a reader can find it elsewhere.

Your response about the representativeness of the practices and the potential for selection bias to be introduced was cogent and thorough. No issues there.

I appreciated your understanding of causal criteria and inference, although (my own biases) your discussion of both plausibility and consistency with the extant literature was not really emphasized in the previous version. I like your thinking and updates ... irrespective of the value of checklist approaches. I don't think I made my point about reverse causation clear enough for you to understand ... and I'll own that limitation of my past review. No changes required from my view, provided your thoughtful points are incorporated.

With respect to having a clear conceptual model and description of your modelling strategy in your methods, I disagree with you that this should be excluded in some form as this is pretty standard for methods sections of articles as robust as yours. And with respect, this was not obvious to me from the titles of the past version of the tables. It was most unclear to me, although I expect that what you did was thoughtful. To me ... this is pretty standard practice ... that at a minimum you would justify your consideration of potential confounders and effect modifiers from the extant literature of from exploratory analyses, and then in your methods section describe the progressive approach to modelling. This was not clear ... perhaps this is just me, but I don't think that this request was unusual.

We can just agree to disagree on the final point ... that when making recommendations for clinical or public health action based on the results, that it would be fairly typical to cite something rigorous in support of those actions. I can see that you have another viewpoint.

I wish you the best, and this was my reaction to your response to my review.

**Reviewer**	**2**
**Name**	**Mustard, Cameron**
**Affiliation**	**Institute for Work and Health**
**Date**	**05-Mar-2026**
**COI**	

With one exception, I am satisfied with the authors' response to my previous review comments.

Perhaps the authors have misunderstood the intent of my suggestion to reference UK national statistics estimating the burden of sickness absence days attributed to work-related health conditions (25% of the total sickness absence days annually among UK workers). The intent of my suggestion was to offer the authors the opportunity to show concordance between the study's estimate that physically demanding work was associated with a PAF of 22.8% and well-established estimates from the Health & Safety Executive that 25% of the total sickness absence days annually among UK workers are attributed to work-related health conditions. I would be satisfied if the authors included a statement of concordance and a reference to HSE estimates in paragraph3 / p10 of the discussion.

Previous review comment

As a final observation, I'd encourage the authors to revisit the discussion of sickness absence attributed to work-related health conditions (p10/line 29). The UK Health & Safety Executive estimates approximately 40 million sickness absence days annually among UK workers, which represents approximately 25% of the total sickness absence days recorded by the UK LFS in 2024 (148 million, all ages). Specifically noting the burden of work-related ill health as a component of population sickness absence would strengthen the manuscript.

Authors response

Thank you for your comment. We do not anywhere refer to "work-related health conditions" in our report (although we do mention work-related risk factors), and we find the concept of "work-related illness" unhelpful. The HSE estimate of 40 million sickness absence days annually in UK workers from such illness presumably is based on reports of absence for illness which the individual considers to be caused or exacerbated by work. However, for most disorders caused by work, the link to occupation cannot be established with confidence in the individual case, even by an experienced clinician with access to good diagnostic facilities. For example, suppose a given exposure to occupational kneeling has been shown epidemiologically to increase the risk of knee osteoarthritis leading to joint surgery with a relative risk of 1.5. It follows that on average, for every three cases with the occupational exposure, one will have developed the disease because of the exposure, and two would have developed it even in the absence of the exposure. However, no-one can say which of the three cases was caused by work.

Our concern in this report is not whether health problems are caused by work, but whether they impinge on capacity to work, leading to high annual sickness absence.

VERSION 2 - AUTHOR RESPONSE

Reviewer: 2
Prof. Cameron Mustard, Institute for Work and Health
Comments to the Author:
With one exception, I am satisfied with the authors' response to my previous review

comments.

Perhaps the authors have misunderstood the intent of my suggestion to reference UK national statistics estimating the burden of sickness absence days attributed to work-related health conditions (25% of the total sickness absence days annually among UK workers). The intent of my suggestion was to offer the authors the opportunity to show concordance between the study's estimate that physically demanding work was associated with a PAF of 22.8% and well-established estimates from the Health & Safety Executive that 25% of the total sickness absence days annually among UK workers are attributed to work-related health conditions. I would be satisfied if the authors included a statement of concordance and a reference to HSE estimates in paragraph3 / p10 of the discussion.

The Labour Force Survey asks participants whether in the last 12-months they have suffered "from any illness, disability or other physical or mental problem that was caused or made worse by your job or by work you have done in the past", and those answering positively who have worked in the past 12 months are asked also how much time they took off work in the last 12 months because of their illness. The illness measured is only that which the individual believes was caused or made worse by work, which as we pointed out in our earlier response, is a subjective assessment and difficult to interpret. Moreover, it makes no reference to what aspect of work is thought to have caused the illness or made it worse. In many cases it may be mental illness ascribed to, or thought to be made worse by, stressful work that is not in the least physically demanding. On the other hand, if illnesses such as heart failure or chronic obstructive airways disease made physically demanding work impossible, it would not be counted if it were not perceived to be caused or made worse by work. In our study the outcome was high annual sickness absence from all types of illness (not just that perceived to be caused or made worse by work), and we examined its association specifically with physically demanding work (not with other aspects of work which might be perceived to cause or exacerbate the illness). Given that the HSE estimate refers to a measure completely different from that assessed by our PAF estimate of 22.8%, we do not think it can legitimately be adduced as supportive of our finding.

We appreciate that the reviewer is trying to be helpful, and we apologise if we did not make our argument clearer in our earlier response.

****
Previous review comment
As a final observation, I'd encourage the authors to revisit the discussion of sickness absence attributed to work-related health conditions (p10/line 29). The UK Health & Safety Executive estimates approximately 40 million sickness absence days annually among UK workers, which represents approximately 25% of the total sickness absence days recorded by the UK LFS in 2024 (148 million, all ages). Specifically noting the burden of work-related ill health as a component of population sickness absence would strengthen the manuscript.

Authors response
Thank you for your comment. We do not anywhere refer to "work-related health conditions" in our report (although we do mention work-related risk factors), and we find the concept of "work-related illness" unhelpful. The HSE estimate of 40 million sickness absence days annually in UK workers from such illness presumably is based on reports of absence for illness which the individual considers to be caused or exacerbated by work. However, for most disorders caused by work, the link to occupation cannot be established with confidence in the individual case, even by an experienced clinician with access to good diagnostic

facilities. For example, suppose a given exposure to occupational kneeling has been shown epidemiologically to increase the risk of knee osteoarthritis leading to joint surgery with a relative risk of 1.5. It follows that on average, for every three cases with the occupational exposure, one will have developed the disease because of the exposure, and two would have developed it even in the absence of the exposure. However, no-one can say which of the three cases was caused by work.

Our concern in this report is not whether health problems are caused by work, but whether they impinge on capacity to work, leading to high annual sickness absence.
****

Reviewer: 1
Prof. William Pickett, Queen's University
Comments to the Author:
Thank you for your detailed and thoughtful response to the issues that I raised upon initial review. I know that it is challenging at times to respond to every nuanced observation made about your work, especially when you are close to it, and I was just trying to provide an honest assessment of its potential strengths and flaws from my viewpoint.

Most of your responses are detailed and thoughtful, and I have respect for your counter-arguments. Here are the remaining ones that didn't sit well with me.

It is not an unreasonable thing to ask for more detail on your study base, and reference to another article for that information ... while technically okay .. puts the onus on the reader to find that information and then also assumes that they have access to that article. I did not think it was an unreasonable thing to ask for, and disagree with your response that a reader can find it elsewhere.

Thank you for your positive comments about the manuscript. As requested, we have now added additional information about the recruitment process through general practices. Please find new text which we have added to the first paragraph of the methods section:

"Briefly, in 2012 the Clinical Practice Research Datalink (CPRD) advertised the HEAF study to all practices in England contributing to its database. Practices that volunteered to assist recruitment into the HEAF study were made known to the research team and all that did so, became foci of recruitment, until the required sample size was reached. In all, 24 general practices contributed to the sampling frame (during January 2013 to June 2014), offering geographical spread with practices from the South, Midlands and North of England. Practices sent an invitation to join the study with an accompanying baseline questionnaire to all men and women born between 1948 and 1964 (other than those suffering from terminal illnesses or recently bereaved).

Your response about the representativeness of the practices and the potential for selection bias to be introduced was cogent and thorough. No issues there.

I appreciated your understanding of causal criteria and inference, although (my own biases) your discussion of both plausibility and consistency with the extant literature was not really emphasized in the previous version. I like your thinking and updates ... irrespective of the value of checklist approaches. I don't think I made my point about reverse causation clear enough for you to understand ... and I'll own that limitation of my past review. No changes required from my view, provided your thoughtful points are incorporated.

Thank you. It has been a useful discussion.

With respect to having a clear conceptual model and description of your modelling strategy in your methods, I disagree with you that this should be excluded in some form as this is pretty standard for methods sections of articles as robust as yours. And with respect, this was not obvious to me from the titles of the past version of the tables. It was most unclear to me, although I expect that what you did was thoughtful. To me ... this is pretty standard practice ... that at a minimum you would justify your consideration of potential confounders and effect modifiers from the extant literature of from exploratory analyses, and then in your methods section describe the progressive approach to modelling. This was not clear ... perhaps this is just me, but I don't think that this request was unusual.

As set out at in the Abstract and Introduction, our aim was to quantify contributions of lifestyle, health-related and employment-related risk factors to high annual sickness absence (HASA) among older workers in England. The risk factors which we examined were selected on the basis of evidence from the extant literature as set out in paragraph 3 of the Introduction. Given that the risk factors analysed were chosen because they were plausible causes of HASA, it was important to adjust for potential mutual confounding, and this was the basis for our analytical strategy. In the case of BMI and smoking, there was a possibility of effects occurring, at least in part, through measured aspects of health that were being analysed as risk factors, and (as we explained in our earlier revision to Table 3) that was taken into account in the modelling. As we indicated previously, the background literature did not suggest any potentially important effect modifiers.

We believe that the modifications that we made previously in response to the reviewer's helpful earlier comments have improved the clarity of the paper, but we remain of the view that a diagrammatic conceptual model would not add usefully to what we now say in the text and table footnotes.

We can just agree to disagree on the final point ... that when making recommendations for clinical or public health action based on the results, that it would be fairly typical to cite something rigorous in support of those actions. I can see that you have another viewpoint.

We agree that unequivocal recommendations for clinical or public health actions should be based on the totality of relevant evidence, and not only on findings from a single study. The only unequivocal recommendation of this sort that we make relates to incapacity from non-specific musculoskeletal pain, and we support that with citation of strong summary evidence - "As at younger ages, strategies to reduce incapacity from non-specific musculoskeletal pain in the back and arm should focus on modifying occupational tasks to make them easier for those with symptoms, while encouraging continued physical activity (36-38). Trials of ergonomic interventions suggest that any benefits in primary prevention are modest (39)."

I wish you the best, and this was my reaction to your response to my review.

Thank you for the substantial time and effort that you have put into reviewing our paper. Your comments have all been helpful and constructive.

Reviewer: 2
If you have selected 'Yes' above, please provide details of any competing interests.: Not

applicable

Reviewer: 1
If you have selected 'Yes' above, please provide details of any competing interests.: Not applicable

---

VERSION 3 - REVIEW

**Reviewer**	**2**
**Name**	**Mustard, Cameron**
**Affiliation**	**Institute for Work and Health**
**Date**	**25-Mar-2026**
**COI**	

I disagree with the authors' perspective. Omitting my suggested amendment leaves the manuscript weakened

---

VERSION 3 - AUTHOR RESPONSE

Reviewer: 2
Prof. Cameron Mustard, Institute for Work and Health
Comments to the Author:
I disagree with the authors' perspective. Omitting my suggested amendment leaves the manuscript weakened

Thank you. We have now added the suggested reference to the Labour Force Survey data, citing it in relation to our proposition that older workers may become increasingly susceptible to injury and illness in physically demanding jobs. Please find new text in the Discussion section. We hope the reviewer will now find the manuscript satisfactory.

"Data from the Labour Force Survey for Great Britain suggest that during 2022-25, an estimated 1.81 days per full-time equivalent worker were lost from work among people aged 55 years and older because of illness or injury which participants believed had been caused or made worse by work, as compared with 1.2 days per person at all ages [33]."
